# *Terminalia albida* treatment improves survival in experimental cerebral malaria through reactive oxygen species scavenging and anti-inflammatory properties

**DOI:** 10.1186/s12936-019-3071-9

**Published:** 2019-12-18

**Authors:** Aissata Camara, Mohamed Haddad, Karine Reybier, Mohamed Sahar Traoré, Mamadou Aliou Baldé, Jade Royo, Alpha Omar Baldé, Philippe Batigne, Mahamane Haidara, Elhadj Saidou Baldé, Agnès Coste, Aliou Mamadou Baldé, Agnès Aubouy

**Affiliations:** 1 UMR152 PHARMADEV, IRD, UPS, Université de Toulouse, Toulouse, France; 2 Institute for Research and Development of Medicinal and Food Plants of Guinea (IRDPMAG), Dubréka, Guinea; 3 Department of Pharmacy, University Gamal Abdel Nasser of Conakry, Conakry, Guinea; 4 Department of Pharmacy, University of Sciences, Technics and Technologies (USTTB) of Bamako, Bamako, Mali

**Keywords:** *Terminalia albida*, Malaria, Experimental cerebral malaria, Inflammatory response, Oxidative stress, UHPLC-HRMS

## Abstract

**Background:**

The development of *Plasmodium* resistance to the last effective anti-malarial drugs necessitates the urgent development of new anti-malarial therapeutic strategies. To this end, plants are an important source of new molecules. The objective of this study was to evaluate the anti-malarial effects of *Terminalia albida*, a plant used in Guinean traditional medicine, as well as its anti-inflammatory and antioxidant properties, which may be useful in treating cases of severe malaria.

**Methods:**

In vitro antiplasmodial activity was evaluated on a chloroquine-resistant strain of *Plasmodium falciparum* (K-1). In vivo efficacy of the plant extract was measured in the experimental cerebral malaria model based on *Plasmodium berghei* (strain ANKA) infection. Mice brains were harvested on Day 7–8 post-infection, and T cells recruitment to the brain, expression levels of pro- and anti-inflammatory markers were measured by flow cytometry, RT-qPCR and ELISA. Non-malarial in vitro models of inflammation and oxidative response were used to confirm *Terminalia albida* effects. Constituents of *Terminalia albida* extract were characterized by ultra‐high performance liquid chromatography coupled with high resolution mass spectrometry. Top ranked compounds were putatively identified using plant databases and in silico fragmentation patterns.

**Results:**

In vitro antiplasmodial activity of *Terminalia albida* was confirmed with an IC50 of 1.5 μg/mL. In vivo, *Terminalia albida* treatment greatly increased survival rates in *P. berghei*-infected mice. Treated mice were all alive until Day 12, and the survival rate was 50% on Day 20. *Terminalia albida* treatment also significantly decreased parasitaemia by 100% on Day 4 and 89% on Day 7 post-infection. In vivo anti-malarial activity was related to anti-inflammatory properties, as *Terminalia albida* treatment decreased T lymphocyte recruitment and expression of pro-inflammatory markers in brains of treated mice. These properties were confirmed in vitro in the non-malarial model. In vitro, *Terminalia albida* also demonstrated a remarkable dose-dependent neutralization activity of reactive oxygen species. Twelve compounds were putatively identified in *Terminalia albida* stem bark. Among them, several molecules already identified may be responsible for the different biological activities observed, especially tannins and triterpenoids.

**Conclusion:**

The traditional use of *Terminalia albida* in the treatment of malaria was validated through the combination of in vitro and in vivo studies.

## Background

Malaria continues to be one of the primary medical concerns in many countries worldwide in terms of number of cases and deaths. In 2017, there were approximately 219 million malaria cases and 435,000 deaths worldwide, 91% of these in sub-Saharan Africa [1]. Today, there are few efficient anti-malarial treatments, and all are derived from artemisinin. However, *Plasmodium falciparum* has developed mechanisms of resistance to artemisinin and its derivatives, particularly in Southeast Asia [2]. Recent studies report increasing time for parasite clearance after treatment in a few parasite isolates originating from West Africa [3, 4]. The development of new treatments based on effective molecules using mechanisms of action, which are different to artemisinin and its derivatives, is thus urgently needed.

Malaria occurs in different forms; it can be uncomplicated, or it may lead to more severe pathologies, particularly cerebral malaria (CM). This is the deadliest form of malaria, with a mortality rate of approximately 15–25% [5]. The cerebral complications are related to a preferential localization of infected erythrocytes (iEs) in the brain through interactions between parasite proteins expressed on the surface of infected red blood cells and brain endothelium [6]. The mechanical obstruction of brain blood flow due to accumulation of iEs and rosetting leads to ischemia, hypoxia and activation of cerebral endothelium [7]. Activated endothelium produces pro-inflammatory cytokines and chemokines involved in the recruitment of immune cells. While the role of monocytes, macrophages and dendritic cells is to remove iEs by phagocytosis, they also produce pro-inflammatory cytokines that activate cytotoxic T cells involved in blood–brain barrier damage [8]. Degradation of haemoglobin by the parasite generates large quantities of toxic free haem and reactive oxygen species (ROS), causing cell damage to the host [9]. In addition, ROS production by monocytes/macrophages is an efficient host defence mechanism, although these oxidative processes lead to an imbalance between pro- and anti-oxidant responses, resulting in oxidative stress [10].

Plants are a significant source of molecules for the development of new drugs. Many plants from traditional medicine have provided opportunities for the identification of anti-malarial molecules. *Artemisia annua* (Asteraceae), a plant used in traditional Chinese medicine, is currently the basis of the last effective treatment strategy [11]. However, specific research of active natural products against severe malaria is scarce. Among the biologically active plant species, *Terminalia albida*, a member of the Combretaceae family traditionally used in Guinea to treat malaria, has shown promising anti-malarial effects against *P. falciparum* (clone Pf-K1, IC50 = 0.6 µg/mL) [12]. It was previously demonstrated that treatment with *Terminalia macroptera*, another species of the same genus, facilitated increased survival in experimental cerebral malaria (ECM) [13]. Therefore, this study was designed to assess the potential anti-malarial and antiplasmodial effects of *Terminalia albida* in the murine model of ECM. Anti-inflammatory and anti-oxidant mechanisms of *Terminalia albida* treatment, which may resolve neuro-inflammation, were also investigated both in vitro and in the murine model of ECM. The phytochemical content of the crude extract was then explored by a dereplication approach in order to understand the origin of the bioactivity.

## Methods

### Plant collection

The stem bark of *Terminalia albida* was collected in Danaya, Préfecture of Dubréka, Guinea. The plant was collected at maturity in a forest area. Authorization for collection of plant materials was obtained prior to collection at the Institut de Recherche et de Développement des Plantes Médicinales et Alimentaires de Guinée (IRDPMAG), Dubréka, Guinea. The identification and authentication was previously carried out by the Department of Botany of IRDPMAG where a voucher specimen (38HK457) was deposited [14].

### Preparation of *Terminalia albida* extract

The stem barks of the plant were shade dried for 2 weeks and ground into powder. The powdered material (600 g) was macerated with 2 L of pure methanol (> 99%) for 72 h. The macerate was then filtered and evaporated under reduced pressure (Buchi^®^ rotary evaporator, model R-200). The dried extracts were stored at − 20 °C until use. Before in vitro and in vivo experiments, stock solutions were dissolved in distilled water to provide working concentrations.

### In vitro antiplasmodial activity

The in vitro antiplasmodial activity of *Terminalia albida* was investigated using the SYBR Green I-based fluorescence assay with *P. falciparum* chloroquine-resistant sensitive strain K1, as described before [15]. Chloroquine was used as the positive control. Briefly, K1 cultures were maintained at 2% haematocrit in RPMI 1640 containing 10% human serum, 3 g/L of glucose, 45 µg/L of hypoxanthine, and 50 µg/L of gentamicin, and incubated at 37 °C under a gas mixture of 5% O_2_, 5% CO_2_, and 90% N_2_. A suspension of sorbitol-synchronized, infected red blood cells was adjusted to 0.5% parasitaemia and 4% haematocrit in complete medium and added to the wells in a 96-well plate. Several wells containing non-parasitized erythrocytes at 4% haematocrit served as reference controls. Stock solutions (at 10 mM in ethanol for plant extracts and in water for chloroquine) were diluted serially in complete medium to test final concentrations ranging from 0 to 10^−5^ M in triplicates in the 96-well plate. Test plates were incubated at 37 °C for 48 h and 100 µL of SYBR Green I in lysis buffer was added to the wells. After 1 h of incubation in the dark at room temperature, fluorescence data were acquired on a Cytofluor II fluorescence multiwell plate reader (PerSeptive Biosystems, Framingham, USA) with excitation and emission wavelength bands at 485 and 530 nm, respectively, and a gain setting equal to 50. After subtraction of background values, the counts were plotted against the logarithm of the drug concentration and curve fitting by nonlinear regression (sigmoidal dose–response/variable slope equation) to yield the 50% inhibitory concentration (IC50) that served as a measure of the anti-malarial activity.

### Animal studies

Female healthy C57BL/6 mice aged 12 weeks and weighing 20–22 g were obtained from Janvier Laboratories (Toulouse, France). For all in vivo experiments described below, the mice were kept in standard and constant laboratory conditions (23–25 °C, relative humidity around 60%, and light/dark cycles, i.e., 12/12 h) with unlimited access to food and tap water. Animal welfare requirements were strictly followed during the experiments as required by the Midi-Pyrénées ethics committee for animal experiments in Toulouse, France. The study was authorized with permit number APAFIS#5921-2016070118008477v3.

### Acute oral toxicity assessment

Acute oral tests were performed as described before with slight modifications [13]. Mice were randomly divided in 2 groups of 3 mice and treated by oral route with a single dose of *Terminalia albida* (2000 mg/kg) or water (20 mL/kg) to follow the limit test proposed by the Organization for Economic Cooperation and Development (OECD) [16]. Animals were observed for the first 4 h after treatment to record immediate deaths, and once daily for 20 days to record deaths and behavioural changes. To evaluate the effect of *Terminalia albida* treatment on body weight, the weight was taken at Days 7, 14 and 20 and compared to Day 0 for both groups (*Terminalia albida* and water).

### In vivo antiplasmodial and anti-malarial effects of *Terminalia albida* in ECM

The rodent malaria parasite, *P. berghei* (strain ANKA) (kindly given by A. Berry, CPTP research unit, Toulouse) was used throughout the study. Mice were infected by intraperitoneal injection with 200 µL of infected blood containing 1 × 10^6^ parasitized erythrocytes on Day 0 (D0). The infected mice were randomly divided into 3 groups of 6 mice and treated by oral gavage for 4 consecutive days with 100 mg/kg of *Terminalia albida* crude extract. The positive control group received chloroquine (5 mg/kg) and the negative control group received distilled water (25 mL/kg). The extracts were administered orally once a day. Weight, parasitaemia, survival and neurological symptoms were followed daily. To evaluate the ability of treatments to prevent weight loss due to infection, the percentages of weight loss as compared to D0 were calculated. Parasitaemia was followed daily through thin blood smears from tail blood from the D3. Blood smears were fixed and stained with a fast-acting variation of May-Grünwald Giemsa staining (RAL 555 kit, RAL Diagnostics). Parasitaemia was determined by light microscopy using a 100 × objective lens as follows:  % parasitaemia = 100 × (number of parasitized RBC/total number of RBC counted). Average percentage of antiparasite activity was calculated at D4, D6, D7 and D8 as [(A − B)/A] × 100 where A is the average percentage parasitaemia in the negative control group (H_2_O) and B is the average percentage parasitaemia in the test group. To monitor the onset of neurological symptoms, 10 parameters were assessed daily between D3 and D7 to determine a rapid murine cerebral behaviour scale (RMCBS) as described by Caroll et al. [17]. The parameters measured were: gait, balance, exploratory behaviour, grooming, body position, limb strength, tactile escape reflex, ear pavilion reflex, toe reflex and aggressiveness. Each parameter was scored 0 to 2, with a 2 score correlating with the highest function. Survival was monitored twice daily. The percentage of survival was determined over a period of 20 days post-infection and compared between groups.

### Brain cell analysis by flow cytometry

After 7 days of infection, mice brains were removed and crushed in PBS−/− (3 mice per group), centrifuged, filtered (100 μM) and mononuclear cells were separated over a Lymphoprep gradient (Alere Technologies AS, Oslo, Norway), and total leucocytes per brain counted. Isolated brain leucocytes were labelled with antibodies coupled to fluorochromes (all from Miltenyi Biotec, France) to distinguish the different T cell populations: CD3-PE (lymphocytes), CD4-VioBright FITC (CD4 T Lymphocytes), CD8-VioGreen (CD8 T Lymphocytes) and Live dead-Violet (dead cells). Cells were collected on a LSR Fortessa cytometer (BD Biosciences) using FACSDiva™ software.

### Gene expression of pro- and anti-inflammatory markers in mice brains

To measure the effect of *Terminalia albida* treatment on the inflammatory response in *P. berghei*-infected mice, the mice were divided into 3 groups of 6 mice, infected and treated with *Terminalia albida* crude extract, chloroquine or distilled water, as before. Mice were sacrificed 7 or 8 days after infection (D7 or D8). Mice brains were dissected and divided longitudinally into two equal parts to be used for RT-qPCR and ELISA experiments. Half of the brain was placed in lysing matrix tubes (MP Biomedicals) containing 650 μL of RLT lysis buffer (from the EZ-10 Spin Column Total RNA Minipreps Super Kit, Bio Basic). The brains were crushed in a FastPrep^®^-24 (MP) and centrifuged for 10 min. mRNA was prepared with the EZ-10 Spin Column Total RNA Minipreps Super Kit (Bio Basic) using the manufacturer’s protocol. Synthesis of cDNA was performed according to the manufacturer’s recommendations (Verso kit, Thermo Scientific). RT–qPCR was performed on a LightCycler 480 system using LightCycler SYBR Green I Master Mix (Roche Diagnostics). The primers (Eurogentec) were designed with the software Primer 3. GAPDH mRNA was used as the invariant control. Serially diluted samples of pooled cDNA were used as external standards in each run for the quantification. The primers used are listed in Table 1.

**Table 1:** Sequences of murine primers used in quantitative RT-PCR experiments

Gene	Sense	Antisense	Product size (bp)
GAPDH	5′-ACA-CAT-TGG-GGG-TAG-GAA-CA	5′-AAC-TTT-GGC-ATT-GTG-GAA-GG	222
IL-1β	5′-GAT-CCA-CAC-TCT-CCA-GCT-GCA	5′-CAA-CCA-ACA-AGT-GAT-ATT-CTC-GAT-G	151
TNF	5′-CTC-CCT-TTG-CAG-AAC-TCA-GG	5′-AGC-CCC-CAG-TCT-GTA-TCC-TT	211
HO-1	5′-CCA-GAG-TGT-TCA-TTC-GAG-CA	5′-CAC-GCA-TAT-ACC-CGC-TAC-CT	174
IL-12	5′-TGG-TTT-GAT–GAT-GTC-CCT-GA	5′-AGG-TCA-CAC-TGG-ACC-AAA-GG	172
IFNg	5′-TGA-GCT-CAT-TGA-ATG-CTT-GG	5′-ACT-GGC-AAA-AGG-ATG-GTG-AC	236
CD11b	5′-AGA-TCG-TCT-TGG-GAG-ATG-CT	5′-GAC-TCA-GTC-AGC-CCC-ATC-AT	169
TLR_2_	5′-TGT-AAC-GCA-ACA-GCT-TCA-GG	5′-TGC-TTT-CCT-GCT-GGA-GAT-TT	196
ICAM-1	5′-AGC-TTG-CAC-GAC-CCT-TCT-AA	5′-AGC-ACC-TCC-CCA-CCT-ACT-TT	159
GranzB	5′-GCT-TCA-CAT-TGA-CAT-TGC-GC	5′-AGA-ACA-GGA-GAA-GAC-CCA-GC	172
NFkB	5′-ACC-GAA-GCA-GGA-GCT-ATC-AA	5′-GCG-TAC-ACA-TTC-TGG-GGA-GT	178
PGEs	5′-CAG-CCT-ATT-GTT-CAG-CGA-CA	5′-CCT-AGG-CTT-CAG-CCT-CAC-AC	157
TGFb	5′-GAC-TCT-CCA-CCT-GCA-AGA-CC	5′-ACG-CGG-GTG-ACC-TCT-TTA G	246
CD36	5′-GAG-CAA-CTG-GTG-GAT-GGT-TT	5′-GCA-GAA-TCA-AGG-GAG-AGC-AC	206
iNOS	5′-ACA-AGG-CCT-CCA-ATC-TCT-GC	5′-TCC-TGG-ACA-TTA-CGA-CCC-CT	95
VEGF	5′-GCT-GTA-ACG-ATG-AAG-CCC-TG	5′-CGC-TCC-AGG-ATT-TAA-ACC-GG	236
*P. berghei*	5′-TCA-TTG-GGC-TCT-CAA-AGG-GT	5′-CAA-TTG-GAG-GGC-AAG-TCT-GG	209

### Levels of cytokines in mice brains

The remaining half of the brain was crushed in PBS between two glass slides and filtered through a 100 µM sterile filter. After centrifugation (1 min at 10,000*g* and room temperature), the remaining pellet was homogenized in 5 mL IGEPAL, a protease inhibitor solution, and stored at − 80 °C until use. The levels of IL-6, TNF, IL-1β, and IL-10 were determined by enzyme-linked immunosorbent assay (ELISA) using commercially available OptiEIA murine kits (BD Biosciences) according to the manufacturers’ instructions.

### Gene expression of pro- and anti-inflammatory markers by murine macrophages after LPS/IFNγ activation and *Terminalia albida* treatment

Peritoneal macrophages were harvested from 6 healthy mice. The peritoneal cavity of each mouse was washed with 5 mL of sterile NaCl 0.9% before harvesting resident peritoneal cells. Collected cells were centrifuged at 1500 rpm for 10 min and the cell pellet was suspended in Dulbecco’s modified Eagle’s medium (Invitrogen) supplemented with glutamine (Invitrogen), penicillin, streptomycin (Invitrogen) and 5% heat-inactivated fetal calf serum. Cells (5.10^5^/well) were left to adhere for 2 h at 37 °C and 5% CO_2_, and non-adherent cells were removed by washing with PBS. After washing, adherent macrophages were immediately stimulated with 10 ng/mL lipopolysaccharide (LPS) and 40 UI/mL IFNγ (Clinisciences) during 2 h. After stimulation, cells were washed and treated with 10 µg/mL *Terminalia albida* crude extract diluted in medium or with medium alone for 4 h at 37 °C and 5% CO_2_. The dose of 10 µg/mL was previously determined by an LDH test showing that this concentration is not toxic to mouse peritoneal macrophages (Additional file 1: Fig. S1). Each condition was tested in triplicate. mRNA extraction, cDNA synthesis and RT–qPCR were performed as described above. The primers used are listed in Table 1.

### Levels of cytokines produced by murine macrophages after LPS/IFNγ activation and *Terminalia albida* treatment

After LPS/IFNγ stimulation (10 ng/mL LPS and 40 UI/mL IFNγ) during 2 h, murine peritoneal macrophages were treated with *Terminalia albida* (10 µg/mL) or medium alone for 24 h at 37 °C and 5% CO_2_. Each condition was tested in triplicate. The supernatants were then assayed to quantify cytokines (TNF, IL-1β, IL-6, IL-10) by ELISA using commercially available OptiEIA murine kits (BD Biosciences) according to the manufacturers’ instructions.

### In vitro ROS production model and *Terminalia albida* scavenging activity

The Light-Up Cell System (LUCS) was used to evaluate the ability of *Terminalia albida* to inhibit the production of ROS by HepG2 cells, as previously described [18]. Briefly, *Terminalia albida* methanolic extract was solubilized in DMSO and 9 serial dilutions were prepared from 1.92 mg/mL to 0.0075 mg/mL. Since DMSO 4% was used to dilute the highest concentration of extract, DMSO 4% was used as negative control. No toxic effect was observed on HEpG2 cells at this concentration. HepG2 cells (75,000/well) were cultured for 24 h in DMEM complemented with 10% FBS and 1% penicillin–streptomycin solution at 37 °C and 5% CO_2_. The experiments were carried out in DMEM without SVF. At least two independent experiments were performed on 96-well plates, each in triplicate. Cells were then incubated for 4 h in the presence or not of increased concentrations of *Terminalia albida* extract. Extract dilutions were added by replacing the media. Cells were then treated with the fluorescent marker thiazole orange at 4 µM for 1 h and fluorescence was measured at 535 nm (emission) during 20 recurrent irradiations at 480 nm (excitation). Antioxidant index (AI) was calculated from the kinetics using the formula AI = 100 – 100 (0∫12 RFUn sample/0∫12 RFUn control). Dose/response curves were obtained by combining AI as a function of the logarithm (10) of the sample concentration and processed by fit sigmoid according to the equation AI = AImin + (AImax − AImin)/(1 + 10^(Log(EC50−CE)*HS)^) to determine EC_50_value.

### UHPLC-HRMS profiling

Acquisition of metabolite profiles of *Terminalia albida* methanol extract (1 mg/mL), data processing and statistical analysis were performed as previously described [19].

### Statistical analysis

Results were expressed as mean ± SEM (standard error of mean) and analysed using Graph Pad Prism software version 6. Comparisons were performed by a one-way analysis of variance (ANOVA) followed by the means multiple comparison method of Bonferroni–Dunnett. Mann–Whitney U-test was used for two-by-two comparisons. Differences were considered significant if P < 0.05.

## Results

### Acute oral toxicity assessment and determination of the test dose

*Terminalia albida* administration to C57BL/6 mice at a dose of 2000 mg/kg did not cause mortality or major behavioural changes among experimental animals during the 20 days of follow-up. Mice body weight was not modified by the administration of *Terminalia albida* (Additional file 2: Fig. S2). Thus, LD50 of *Terminalia albida* is greater than 2000 mg/kg in C57BL/6 mice. *Terminalia albida* crude extract, as prepared in this work, can therefore be classified as category 5 and considered non-toxic orally, according to the OECD’s Globally Harmonized System of Classification [16]. Based on these results, anti-malarial activity in ECM was assessed at 100 mg/kg.

### In vitro and in vivo antiplasmodial activity

**Fig. 1:**
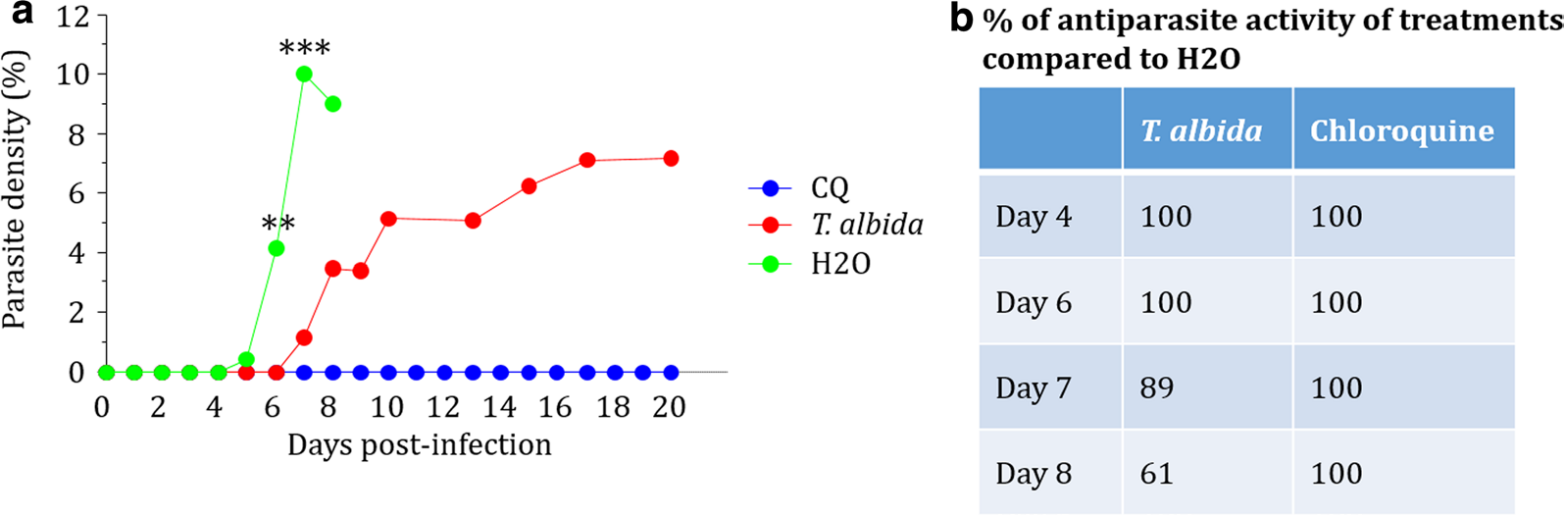
Antiplasmodial activity of *Terminalia albida* treatment in ECM model. C57BL/6 mice were infected with *P. berghei* ANKA and treated 2 h later with chloroquine (5 mg/kg), *Terminalia albida* crude extract (100 mg/kg) or water (25 mL/kg) from Day 0 to Day 3. **a** Mean parasite densities during infection. **b** Percentages of parasite suppression according to the treatment, calculated by comparison to H_2_O treated mice. **P < 0.005, ***P < 0.0005 compared *Terminalia albida* to H_2_O group

*Terminalia albida* crude extract presented an IC50 of 1.5 μg/ml and IC50 for chloroquine was 0.08 µM on the chloroquine-resistant K1 strain of *P. falciparum*. In vivo, mice that received water were parasitized from D4, while mice treated with *Terminalia albida* were positive from D7. Mice that received water were all dead at D8. At D4, parasite chemosuppression by *Terminalia albida* was 100% and this complete inhibition lasted until D6. At D7, *Terminalia albida* treatment resulted in parasite suppression of 89% compared to water (Fig. 1a, b, P < 0.0001). However, *Terminalia albida* treatment did not limit parasite multiplication since parasitaemia increased until 7% at D20. Conversely, chloroquine treatment abolished parasite multiplication (Fig. 1a, b).

### Effect of *Terminalia albida* treatment on survival, weight and neurologic symptoms in ECM

**Fig. 2:**
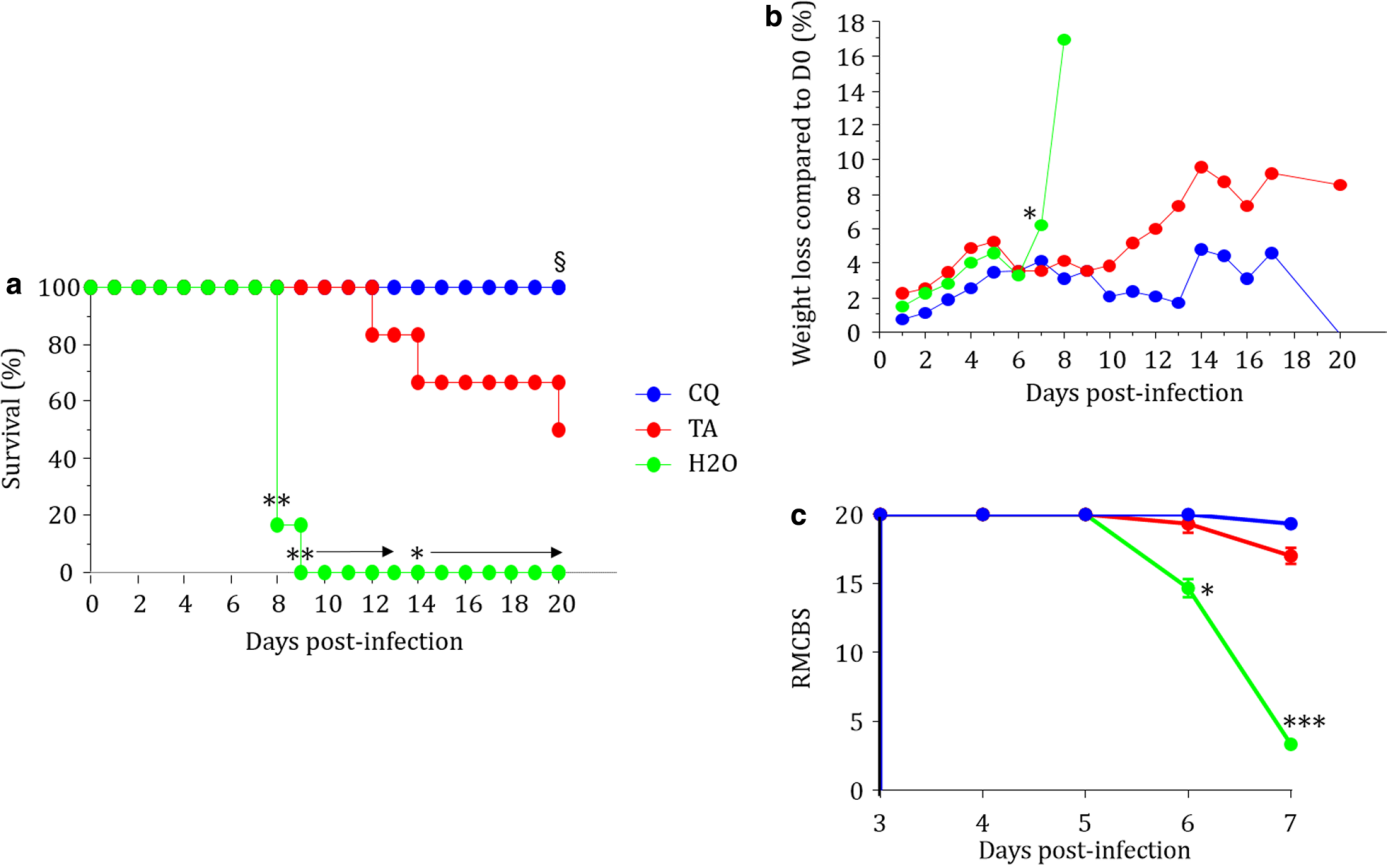
Effect of *Terminalia albida* treatment on survival, weight and cerebral symptoms in ECM model. C57BL/6 mice were infected with *P. berghei* ANKA and treated 2 h later with chloroquine (5 mg/kg), *Terminalia albida* crude extract (100 mg/kg) or water (25 mL/kg) from Day 0 to Day 4. **a** Percentage of survival. **b** Percentage of weight loss at each day compared to Day 0. **c** Rapid murine cerebral behavior scale (RMCBS) between D3 and D7. *P < 0.05 compared *Terminalia albida* to H_2_O groups at D7. *P < 0.05 and **P < 0.005 compared *Terminalia albida* to H_2_O groups. ^§^P < 0.05 compared *Terminalia albida* to chloroquine groups

*Terminalia albida* treatment greatly improved survival as chloroquine and *Terminalia albida* treatments were statistically similar until D20 although Fig. 2a suggests that *Terminalia albida* treatment was less effective than chloroquine from D12 onwards. At D20, chloroquine was statistically more effective than *Terminalia albida* treatment (Fig. 2a, P = 0.045). *Terminalia albida* treatment prevented death up to D12 and maintained a 50% survival rate at D20 (Fig. 2a. P = 0.003 at D8, P = 0.0005 from D9 to D13, and P < 0.05 until D20 for the comparison between *Terminalia albida* and water). *Terminalia albida* treatment improved the overall condition of the mice since the treated group lost less weight than the water group (the groups were compared up to D7 only because the group that received water had only one living mouse left on D8. P = 0.037 at D7. Figure 2b). The comparison of RMCBS between groups showed a rapid deterioration of cerebral functions in untreated mice from D5 (Fig. 2c). Although mice treated with *Terminalia albida* had slightly impaired brain functions at D6 and D7, there was no statistical difference between the scores of the CQ and *Terminalia*-treated mice at D6 and D7 (Fig. 2c).

### Effect of *Terminalia albida* on T cell infiltration in the brain of *Plasmodium berghei*-infected mice

**Fig. 3:**
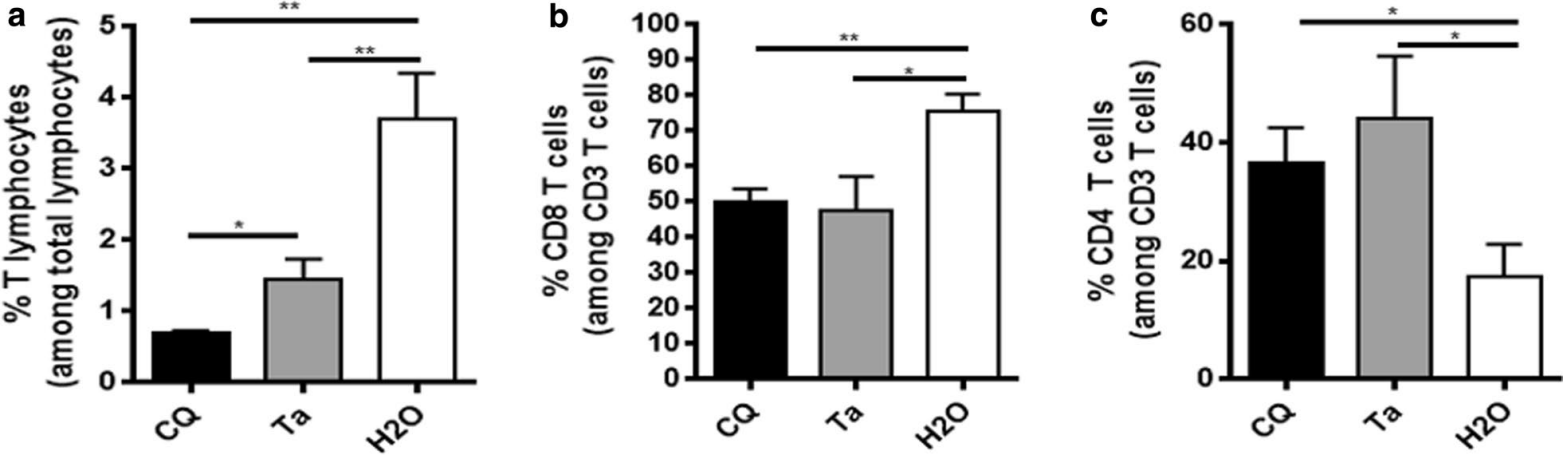
Effect of *Terminalia albida* on cell infiltration in the brain of *Plasmodium berghei*-infected mice. C57BL/6 mice were infected with *P. berghei* and treated 2 h later with chloroquine (5 mg/kg), *Terminalia albida* crude extract (100 mg/kg) or water (25 mL/kg) from Day 0 to Day 4. Brains were analysed by flow cytometry at D7 post-infection. Percentages of cell populations were compared between groups for CD3^+^ (**a**), CD8^+^ (**b**) and CD4^+^ T lymphocytes (**c**). *P < 0.05, **P < 0.005, ***P < 0.0005

Leucocyte recruitment and activation in the brain is one of the key mechanisms of ECM, particularly CD8 effector T cells [20]. To assess such mechanism of *P. berghei*-infected mice according to the treatment received (chloroquine or *Terminalia albida* or H_2_O), the percentages of the different T cell populations were compared in brains after 7 days of infection by flow cytometry. Interestingly, the percentages of CD3 T and CD3 CD8 T cells in brain was lower in mice treated with chloroquine or *Terminalia albida* extract, compared to mice that received water (Fig. 3a, b). Conversely, the percentage of CD4 T cells was higher in chloroquine and *Terminalia albida*-treated mice, compared to water-treated mice (Fig. 3c, P = 0.04 for both comparisons).

### Absence of pro-inflammatory response in brains of *Terminalia albida*-treated mice during ECM

**Fig. 4:**
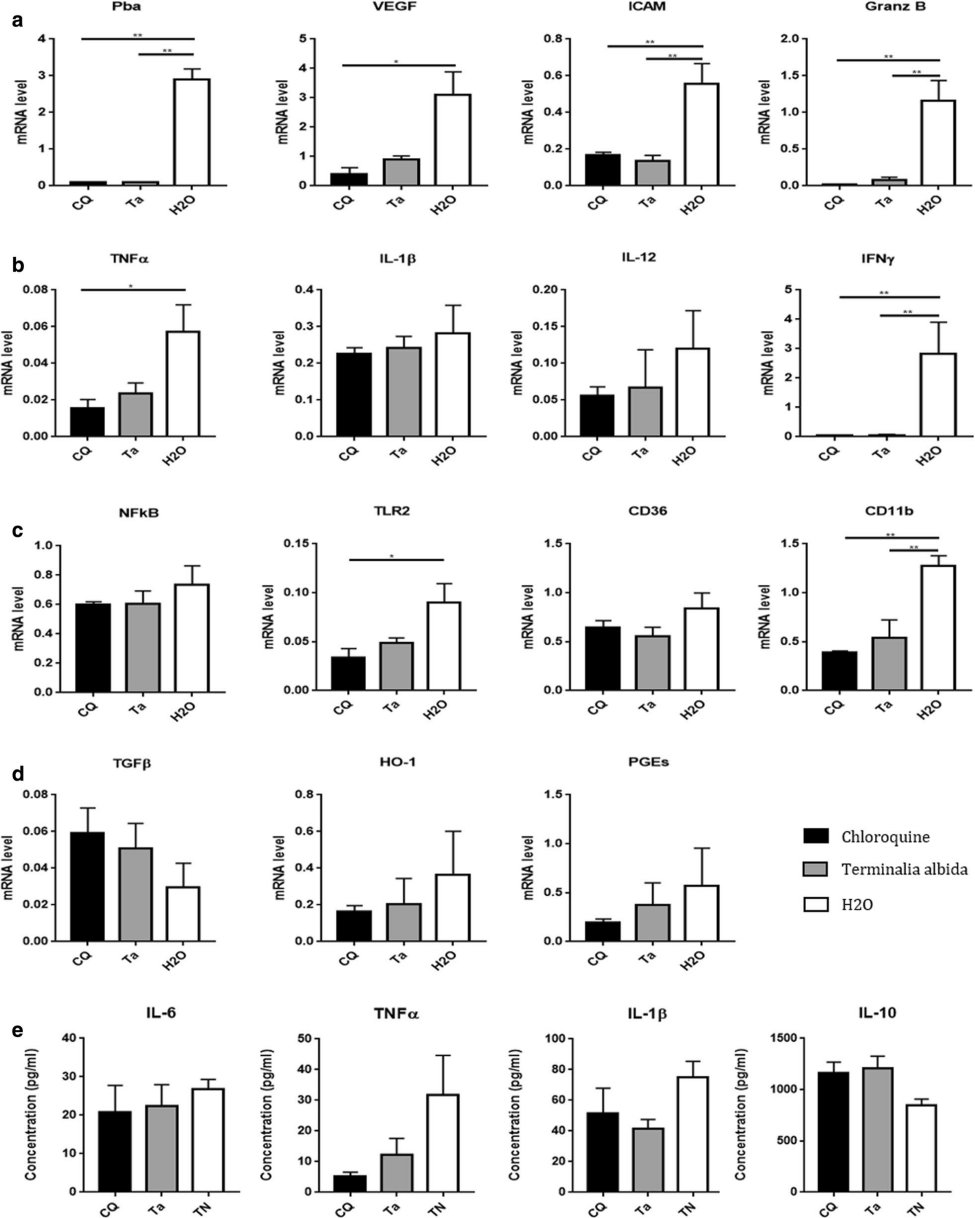
Effect of *Terminalia albida* treatment on the expression of pro- and anti-inflammatory markers in brains of *Plasmodium berghei*-infected mice. C57BL/6 mice were infected with *P. berghei* and treated with chloroquine (5 mg/kg), *Terminalia albida* (100 mg/kg) or water. Brains were harvested at D7 (for *P. berghei* and VEGF) or D8 post-infection (for the other markers). Gene expression was measured by RT-qPCR and cytokine levels were measured by ELISA. Gene expression levels of (**a**) *P. berghei*, VEGF, ICAM and Granzyme B; **b** pro-inflammatory cytokines, **c** other pro-inflammatory markers, and **d** anti-inflammatory markers. **e** Cytokine levels in pg/mL. *P < 0.05, **P < 0.005, ***P < 0.0005. Data presented are mean ± standard deviation

To assess the immunological state of brains from *Terminalia albida*-treated mice during ECM, mRNA levels of pro- and anti-inflammatory markers were measured in the 3 groups of mice (*Terminalia albida*, chloroquine and H_2_O). *Plasmodium berghei* mRNA was undetectable at D7 in brains of chloroquine and *Terminalia albida*-treated mice (Fig. 4a). The expression of VEGF, a marker of vascular permeability and endothelial activation, was lowered in the chloroquine group compared to the water group (Fig. 4a). Conversely, the expression of ICAM-1, another marker of endothelial activation was diminished in brains of mice treated with *Terminalia albida* (Fig. 4a, P = 0.002). Among the pro-inflammatory cytokines tested, IFNγ expression was completely abolished in mice treated with *Terminalia albida* similarly to the chloroquine group, in comparison to untreated mice (Fig. 4b. P = 0.006 and P = 0.005 for *Terminalia albida versus* H_2_O and chloroquine *versus* H_2_O, respectively). Interestingly, *Terminalia albida* treatment also significantly limited the expression of granzyme B similarly to chloroquine (Fig. 4a. P = 0.003 and 0.002 for H_2_O *versus Terminalia albida* and *versus* chloroquine). For TNF, IL-1β and IL-12 expression, the effect of *Terminalia albida* treatment compared to chloroquine or H_2_O was not significant (Fig. 4b). Among the other pro-inflammatory markers tested, *Terminalia albida* treatment also diminished CD11b expression similarly to chloroquine (Fig. 4b, P = 0.006). Finally, for TLR2, NFκB, PGES and anti-inflammatory markers (TGFβ, CD36 and haem oxygenase 1 named HO-1), *Terminalia albida* treatment had no significant effect (Fig. 4c, d). Cytokine levels were also measured in brains on D8. However, cytokine levels were similar regardless of the treatment received by the mice (Fig. 4e).

### Anti-inflammatory properties of *Terminalia albida* in a non-malarial context

**Fig. 5:**
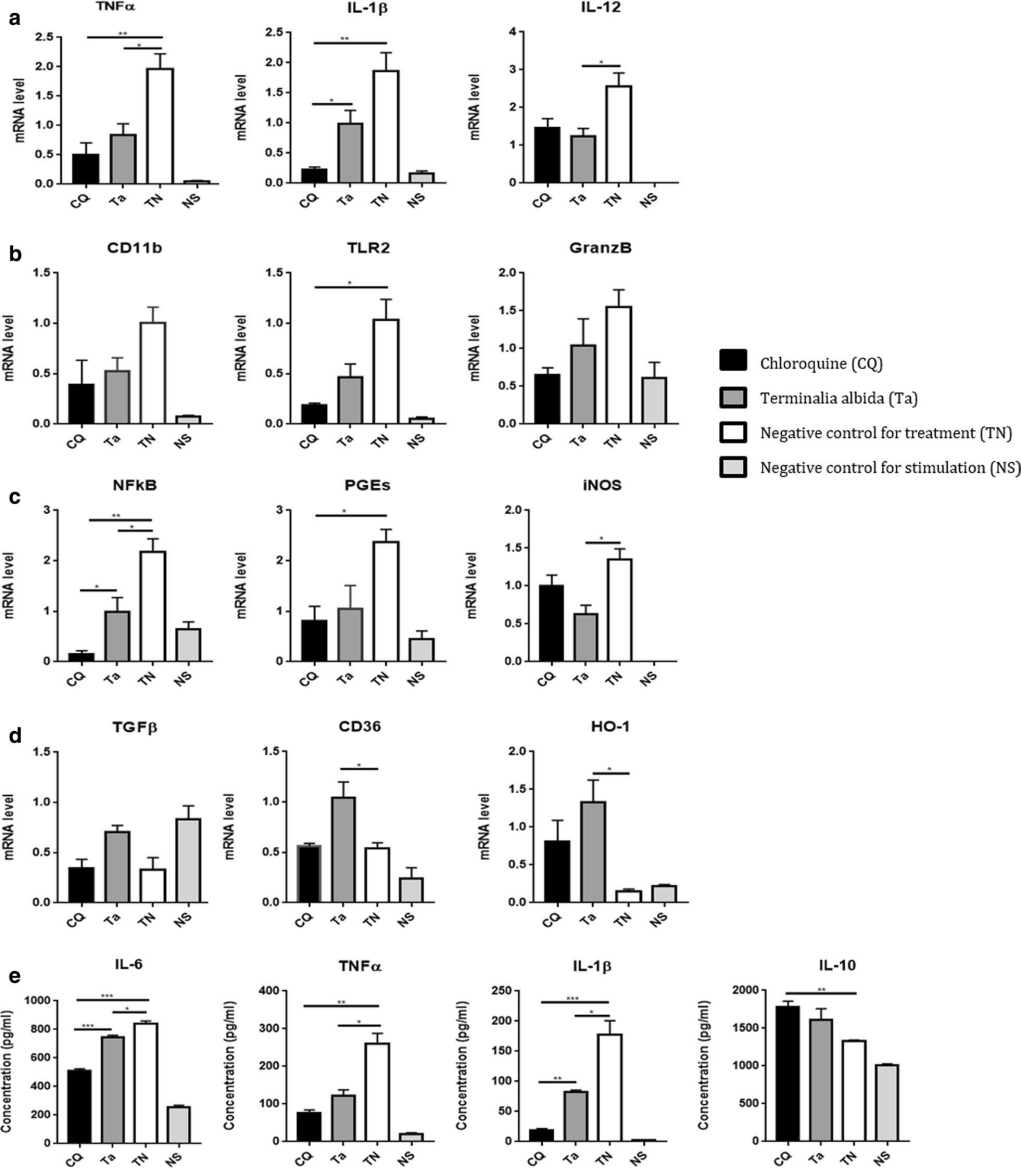
Assessment of in vitro anti-inflammatory properties of *Terminalia albida.* Murine macrophages were activated by LPS and IFNγ in the presence or not of 10 µg/mL of *Terminalia albida*. Gene expression levels of **a** pro-inflammatory cytokines, **b** other pro-inflammatory markers, **c** pro-inflammatory signalling pathways, and **d** anti-inflammatory markers. **e** Cytokine levels measured in supernatants, in pg/mL. *P < 0.05, **P < 0.005, ***P < 0.0005. Data are presented as mean ± SD

To further confirm the anti-inflammatory properties of *Terminalia albida* assessed in the ECM model, additional experiments were performed by stimulating the peritoneal macrophages of C57BL/6 healthy mice with LPS and IFN and treating them with the methanolic extract of *Terminalia albida*. The expression of several pro-inflammatory markers was decreased following treatment of the cells with *Terminalia albida* compared to untreated cells: TNF (P = 0.04), IL12 (P = 0.003), NFkB (P = 0.005) and inducible nitric oxide synthase (iNOS) (P = 0.001) (Fig. 5a, c). Notably, CD36 and HO-1, two anti-inflammatory markers, were more expressed by *Terminalia albida*-treated cells (Fig. 5d, P = 0.001 and P = 0.01, respectively). In addition, a significant decrease was also obtained in the enzymatic quantification of TNF, IL-6 and IL-1β for *Terminalia albida* treated-cells (Fig. 5e, P = 0.001, P = 0.02 and P = 0.03, respectively).

### Anti-oxidative properties of *Terminalia albida*

**Fig. 6:**
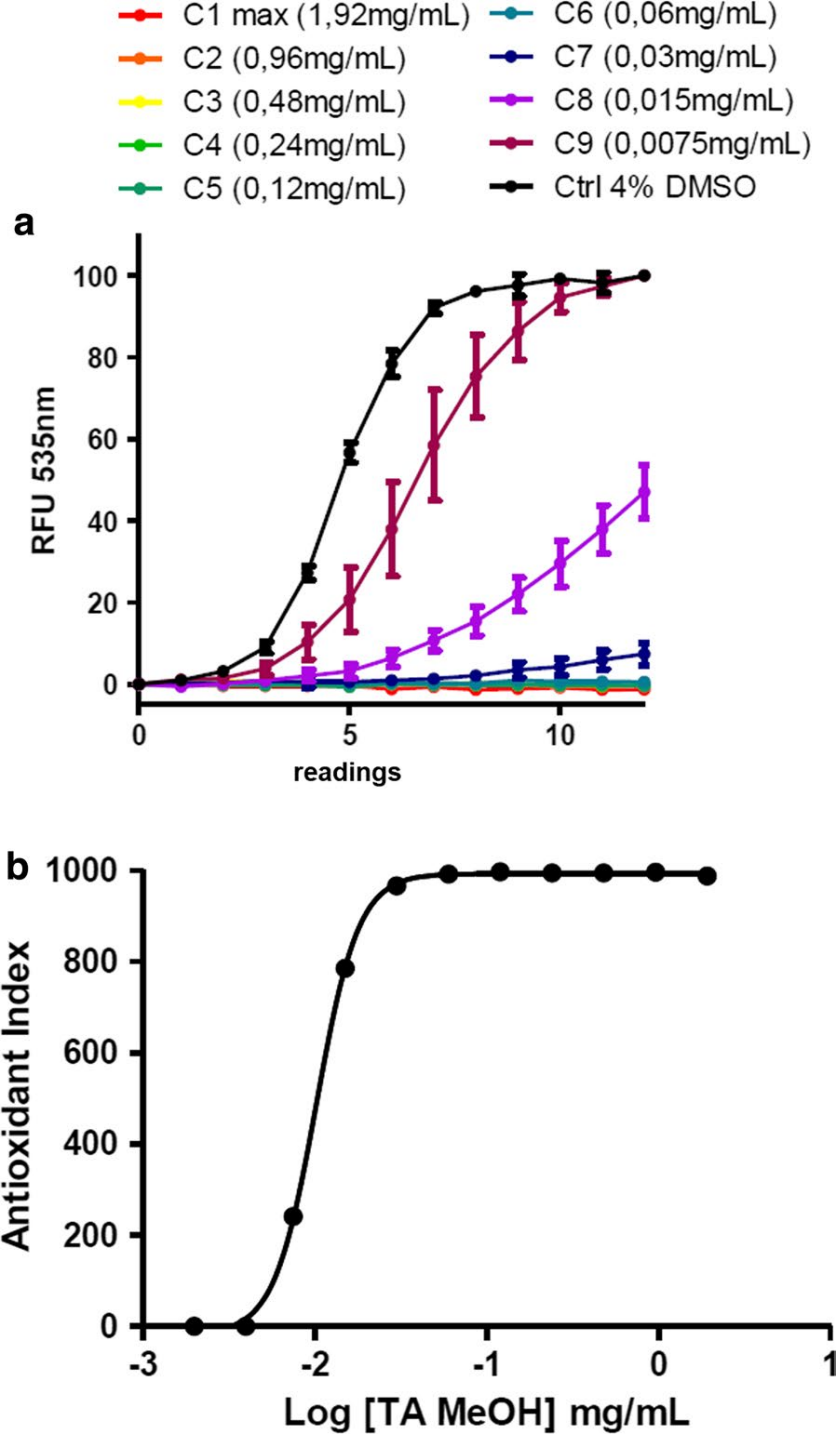
LUCS anti-oxidant assay. **a** Kinetics of fluorescence emission recorded under illumination for different concentrations of extract (7.5 10^−3^ mg/mL–1.92 mg/mL) using the LUCS assay. **b** Corresponding antioxidant index calculated from RFU values as follows: AI = 1000–1000 (0∫12 RFUn sample/0∫12 RFUn control)

The evaluation of *Terminalia albida*’s ability to neutralize intracellular radical species of the human HepG2 cells showed a remarkable dose-dependent antioxidant activity (Fig. 6a). The antioxidant index (AI) calculated from the area under curves was 997 at concentrations greater than or equal to 60 μg/mL (Fig. 6b). The EC50 of the extract was 10.2 μg/mL. Finally, the absence of a fluorescent signal higher than the control at time 0 indicates an absence of cytotoxicity at 4 h of treatment.

### Phytochemical characterization of the *Terminalia albida* crude extract

After maceration with methanol for 72 h, 253.5 g of crude extracts were obtained. The extracts were qualitatively analysed by UHPLC-HRMS. Metabolite profiling of *Terminalia albida* was acquired in positive and negative ionization mode. Qualitative analysis by UHPLC-HRMS of *Terminalia albida* stem bark enabled the putative identification of 16 known compounds through HRMS and MS/MS fragmentation patterns using MzMine, MS-DIAL and MS-FINDER software (Table 2). Among the top 12 annotated compounds, 6 are tannins (flavogallonic acid, flavogallonic acid-Me ester, ellagic acid, ellagic acid 3,8-di-Me ether, corilagin, ellagic acid; 2,3,7-tri-me ether), 4 are triterpenoids (23-O-galloylarjunolic acid 28-O-β-d-glucopyranosyl ester, 23-galloylarjunolic acid, 23-galloylterminolic acid, 7-beta-hydroxy-23-deoxojessic acid), 1 is phenol glucoside gallate (vanillic acid 4-(6-galloylglucoside)) and 1 is cycloartanols and derivatives (ouadrangularic acid F).

**Table 2:** Putative identified features (m/z × RT pairs) using HRMS and MS/MS fragmentation patterns using MzMine, MS-finder and DNP database

ID	RT (min)	*m/z*	Formula finder	Δ Da	Putative ID	Ontology	Scoring
1	3.59	481.0977 [M–H]^−^	C_21_H_22_O_13_	0.0010643	Vanillic acid 4-(6-galloylglucoside)	Phenolic glucoside gallate	7.7481
2	4.11	447.0561 [M–H]^−^	C_20_H_16_O_12_	0.0007995	Eschweilenol C	Hydrolyzable tannins	7.5965
3	5.57	329.0296 [M–H]^−^	C_16_H_10_O_8_	0.0006908	Ellagic acid-3,8-Di-Me ether	Hydrolyzable tannins	7.5676
4	3.08	633.0719 [M–H]^−^	C_27_H_22_O_18_	0.0014374	Corilagin	Hydrolyzable tannins	7.5558
5	4.27	300.9984 [M–H]^−^	C_14_H_6_O_8_	0.0078670	Ellagic acid	Hydrolyzable tannins	7.4150
6	5.90	801.405 [M–H]^−^	C_43_H_62_O_14_	0.0016802	23-O-Galloylarjunolic acid 28-O-β-d-glucopyranosyl ester	Triterpenoids	7.3505
7	3.97	483.0196 [M–H]^−^	C_22_H_12_O_13_	0.000914	Flavogallonic acid-Me ester	Hydrolyzable tannins	7.3463
8	2.69	469.0038 [M–H]^−^	C_21_H_10_O_13_	0.0010639	Flavogallonic acid	Hydrolyzable tannins	7.1290
9	7.26	639.3527 [M–H]^−^	C_37_H_52_O_9_	0.0011568	23-Galloylarjunolic acid	Triterpenoids	7.0819
10	6.92	655.3481 [M–H]^−^	C_37_H_52_O_10_	0.0006714	23-Galloylterminolic acid	Triterpenoids	6.9868
11	10.7	501.3589 [M–H]^−^	C_31_H_50_O_5_	− 0.0003518	7-beta-Hydroxy-23-deoxojessic acid	Triterpenoids	6.7122
12	7.71	547.3273 [M–H]^−^	C_31_H_48_O_8_	0.000342	Quadrangularic acid F	Cycloartanols and derivatives	6.7025

## Discussion

In this study, the anti-malarial and antiparasite activity of *Terminalia albida* found respectively in ECM and in vitro was high. In vivo, mice survived until D12, the limit for neurological symptoms in this model, and there was a 50% rate after 20 days of infection, while untreated mice were all dead by D8. Such a result obtained with a crude plant extract is quite remarkable when compared to many other similar studies (performed with crude plant extracts at doses ≤ 300 mg/Kg) in which mean mice survival often did not exceed 9 days [21–23]. Antiparasite activity was also very high in vivo, 100% at D4 and until D6, 89% at D7, and 61% at D8. Interestingly, mice treated with *Terminalia albida* have developed a parasitaemia that remained below 8% until D20, while the average parasitaemia of untreated mice reached 10%. This result may be explained by the pharmacokinetics of the active principle(s) responsible for the antiparasite activity. Further bio-guided fractionation is needed to decipher the reasons of such activity.

In addition, antiparasite activity found in vitro was also interesting with an IC50 of 1.5 µg/ml. This value is slightly higher than that of 0.6 µg/mL, previously found by Traore et al. in 2014 [12]. This difference may be due to the disparity of harvesting locations, harvest time, and extraction methods that influence plant biological active compounds [24]. In addition, Traore et al. used the lactate dehydrogenase assay to measure antiplasmodial activity whereas a Sybr green assay was used in the present study. These different methods may also explain the small disparities between the two studies.

In this research, the aim was to elucidate whether, in addition to its antiparasitic properties, *Terminalia albida* presents anti-inflammatory and anti-oxidant activities useful for the resolution of cerebral malaria. During malaria, the first immune responses (oxidative and inflammatory) induced by monocytes are essential to control parasite multiplication. However, excessive and inappropriate activation of the immune system is detrimental to the host and contributes to the severe form that can lead to death [25]. Mechanisms leading to ECM involve the recruitment of T lymphocytes to the brain, particularly CD8 T cells known to be responsible for lethal neuropathology [8]. In the ECM model, treatment with *Terminalia albida,* similarly to chloroquine, greatly restrained the recruitment of T cells and more specifically of CD8 T cells in mice brains after 7 days of infection. It seems logical since at D7, the parasitaemia of the treated mice was very low and the parasite mRNA undetectable.

Here, brains from infected mice treated with the methanolic extract of *Terminalia albida* showed reduced expression of IFNγ, CD11b, ICAM-1 and granzyme B. The secretion of IFNγ by NK cells contributes to the CM pathogenesis by increasing the expression of endothelial receptors including ICAM-1 and VCAM involved in the sequestration of parasitized red blood cells in the murine model [26]. This phenomenon induces the activation of endothelial cells by excessive production of pro-inflammatory cytokines such as TNF, IL-6, IL-1β, IL-12 and the recruitment of inflammatory leukocytes in the cerebral micro-vessels that damage the blood–brain barrier [25]. CD11b constitutes a marker of brain endothelium disruption, as described before [27]. In addition, CD8 + T cells have been identified as a major mediator in death during ECM by their capacity to produce granzyme B able to kill *P. berghei* antigen-presenting endothelial cells and to damage neuronal cells [8]. Thus, during ECM, *Terminalia albida* treatment not only limits parasitaemia but also reduces the expression of mediators involved in pathogenesis. Such anti-inflammatory properties were confirmed in vitro in a non-malarial context. After LPS/IFNγ stimulation in murine peritoneal macrophages, *Terminalia albida* treatment led to a significant inhibition of the expression of TNF, IL-1β, IL-6 and IL12, four pro-inflammatory cytokines. NFkB, the transcription factor regulating the inflammatory response has been suggested as therapeutic target to treat severe malaria [28]. In the present study, NFkB was also less expressed by cells treated with the plant compared to untreated cells. Similarly, CD36, an important marker for malaria, was higher in treated cells compared to untreated ones (Fig. 4). Through PPARγ activation, the scavenger receptor CD36 is known for its implication in *P. berghei* elimination through non-opsonic phagocytosis [29].

*Terminalia albida* also presented highly interesting antioxidant properties in vitro. LPS/IFNγ-activated cells treated with *Terminalia albida* showed a lower expression of iNOS and higher HO-1. In addition, *Terminalia albida* neutralized intracellular radical species of the human HepG2 cells in a dose-dependent antioxidant activity. It is largely accepted that oxidative stress is involved in the pathogenesis of severe malaria [30]. Nitric oxide is produced via the enzyme NOS and the substrate l-arginine. However, its beneficial or unfavourable effect is controversial in the experimental model [31, 32]. In the present study, *Terminalia albida* limits iNOS expression in vitro and increases survival in ECM. HO-1 is a key protective gene in the host infected by *Plasmodium,* able to catabolize toxic free haem into iron, biliverdin and carbon monoxide. HO-1 induction has been shown to prevent blood–brain barrier disruption, brain microvasculature congestion and neuro-inflammation in the murine model [33]. Thus, *Terminalia albida* presents antiplasmodial, anti-inflammatory and anti-oxidant properties. However, these activities are not sufficient to limit parasitic multiplication as chloroquine does. These results suggest that the anti-inflammatory and anti-oxidant activities of the extract limit neuro-inflammation secondary to infection, but that the antiplasmodial activity is not sufficient to cancel the multiplication of the parasite in this model.

*Terminalia* species are rich sources of secondary metabolites including cyclic triterpenes and their derivatives, and polyphenols (flavonoids, phenolic acids and tannins). Several *Terminalia* extracts or fractions have been tested for their anti-*Plasmodium* activities [34–42] but most of these studies lack chemical characterization of the studied extracts, except Muganga et al. [36] who isolated the active compounds, through a bioguided fractionation [36]. Among all the compounds isolated, ellagic acid, already know to have a strong anti-malarial activity (IC50 between 90 and 175 ng/mL) [43], was found to be the main antiplasmodial compound (IC_50_ = 0.175 μg/mL) in *Terminalia mollins*, while ellagic acid derivatives were inactive, suggesting the crucial role of the free hydroxyl groups in antiplasmodial activity. In addition, gallic acid and some condensed tannins, such as catechin, gallocatechin and epigallocatechin, have been isolated and found to have a lower antiplasmodial activity (IC50 > 25 μg/mL) than the one found for ellagic acid, and a mixture of punicalagin A and B (anomeric isomers) did not present any interesting antiplasmodial activity. Akanbi et al. [44] evaluated the anti-malarial activity of total saponins extracted from *Terminalia avicennioides* and concluded that the *Terminalia*’s saponins may be able to increase the level of immunity against malaria infection, responsible for the antiplasmodial activity displayed by the extract [44]. However, saponin isolation was based on a phytochemical extraction followed by TLC analysis and a non-specific colorimetric test. No complementary analyses such as IR, UV, LC–MS/MS dereplication or NMR, were carried out to confirm the composition of this extract, which makes these results questionable. In addition, saponins are characterized by a strong haemolytic activity and may be toxic [45]. Finally, 23-galloylarjunolic acid, a triterpene of the galloyl group type, has shown interesting antiplasmodial activity against the chloroquine-sensitive (D6) and chloroquine-resistant (W2) strains with respective IC50s of 4.5 µg/mL and 2.8 µg/mL and selectivity index greater than 1 [46]. Thus, in *Terminalia albida* extract, ellagic acid and 23-galloylarjunolic acid may be responsible, at least in part, for the antiplasmodial activity.

Phenolic compounds are widely described in the literature for their potential biological activity such as anti-inflammatory and immunomodulatory effects [47, 48]. They are excellent anti-oxidants due to the presence of a hydroxyl group capable of capturing oxygen free radicals [49]. In addition to anti-*Plasmodium* activity, ellagic acid has known anti-oxidant and anti-inflammatory activity that could explain the higher survival rate obtained after *Terminalia albida* treatment in ECM [47, 50]. In a model of carrageenan-induced inflammation, ellagic acid prevented the production of pro-inflammatory mediators and promoted the production of anti-inflammatory IL-10 and antioxidant glutathione [51]. Eschweilenol C has also been reported for anti-inflammatory activity in the aqueous extract of *Terminalia fagifolia* by inhibition of NFkB pathway in LPS-activated microglial cells [48]. Corilagin has also been studied for its anti-inflammatory activity based on the inhibition of the NFkB signaling pathway and even tested as a treatment in a model of sepsis [52–54]. Anti-inflammatory and anti-oxidant effects of a derivative of vanillic acid, vanillic acid 4-(6-galloylglucoside), were also reported both in vitro and in vivo in the carrageenan-based inflammation murine model [55, 56]. Finally, cyclic triterpenes are also a phytochemical group with various biological activities, particularly in inflammatory and oxidative diseases [57]. Nevertheless, further bioassay-guided fractionation will be necessary to confirm the origin of the antiplasmodial, anti-inflammatory and anti-oxidant activities of *Terminalia albida*, including synergistic potential between tannins, lignans and terpenoids found in this plant.

## Conclusion

*Terminalia albida* displays highly interesting anti-malarial, anti-inflammatory and anti-oxidant properties. UHPLC-HRMS analysis showed that 12 compounds are found in *Terminalia albida* and may be implicated in its biological activities. However, further investigations into the long-term toxicity, prophylactic effects and antiparasite mechanisms are necessary before recommending the use of *Terminalia albida* or its constituents for malaria treatment or prevention.

## Supplementary information

**Additional file 1: Fig. S1.** *Terminalia albida* cytotoxicity against healthy murine peritoneal macrophages by the lactate deshydrogenase (LDH) test. Cells (2.10^5^/well) were left to adhere for 2 h at 37 °C and 5% CO2, and non-adherent cells were removed by washing with PBS. Adherent cells were treated with serial dilutions of *Terminalia albida* extract (100 μg/mL, 50 μg/mL, 25 μg/mL, 10 μg/mL, 5 μg/mL) and incubated at 37 °C and 5% CO2 for 24 h. LDH leakage from the cells was determined using a commercial LDH cytotoxicity detection kit according to the manufacturer’s protocols (Cytotoxicity detection Kit, Roche, France). Absorbance was measured at 490 nm with a Wallac Victor 2 1420 Multilabel Counter. Cell mortality was calculated as a percentage: (absorbance of wells treated with extract *100/absorbance of wells treated with triton).

**Additional file 2: Fig. S2**. Acute oral toxicity test in vivo: effect of *Terminalia albida* treatment on body weight. Mice were treated by oral route with a single dose of *Terminalia albida* (2000 mg/kg) or water (20 mL/kg). To evaluate the effect of *Terminalia albida* treatment on body weight, the weight was taken at D7, D14 and D20 and compared to D0 for both groups (*Terminalia albida* and water).

## Data Availability

The datasets used and/or analysed during the current study are available from the corresponding author on reasonable request.

## Abbreviations

CD: cluster of differentiation
CPTP: Centre de Physiopathologie de Toulouse Purpan
ECM: experimental cerebral malaria
HepG2: liver hepatocellular cells G2
HO-1: haem oxygenase-1
IC50: 50% inhibitory concentration
ICAM-1: intercellular adhesion protein 1
iEs: infected erythrocytes
IFNγ: interferon gamma
IL: interleukin
iNOS: nitric oxide synthase
LC/MS: liquid chromatography–mass spectrometry
LD50: 50% lethal dose
LPS: lipopolysaccharide
NFkB: nuclear factor-kappa B
NK: natural killer
OECD: Organization for Economic Cooperation and Development
PPARγ: peroxisome proliferator-activated receptor
ROS: reactive oxygen species
TNF: tumor necrosis factor
VCAM-1: vascular cell adhesion protein 1
