# Supplementary figures and images for: Terminalia albida treatment improves survival in experimental cerebral malaria through reactive oxygen species scavenging and anti-inflammatory properties
Source: Malar J. 2019 Dec 18;18:431. doi: 10.1186/s12936-019-3071-9 (PMC6921526; doi:10.1186/s12936-019-3071-9)

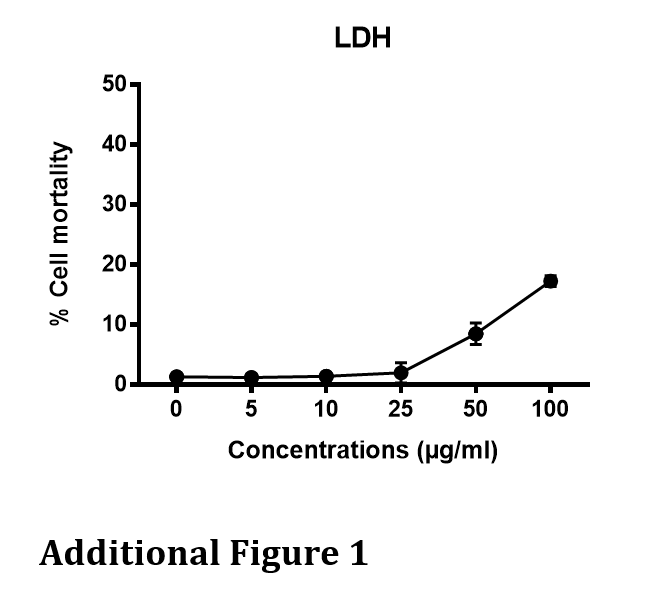

Supplement: Supplementary file 1 — Additional file 1: Fig. S1. Terminalia albida cytotoxicity against healthy murine peritoneal macrophages by the lactate deshydrogenase (LDH) test. Cells (2.105/well) were left to adhere for 2 h at 37 °C and 5% CO2, and non-adherent cells were removed by washing with PBS. Adherent cells were treated with serial dilutions of Terminalia albida extract (100 μg/mL, 50 μg/mL, 25 μg/mL, 10 μg/mL, 5 μg/mL) and incubated at 37 °C and 5% CO2 for 24 h. LDH leakage from the cells was determined using a commercial LDH cytotoxicity detection kit according to the manufacturer’s protocols (Cytotoxicity detection Kit, Roche, France). Absorbance was measured at 490 nm with a Wallac Victor 2 1420 Multilabel Counter. Cell mortality was calculated as a percentage: (absorbance of wells treated with extract *100/absorbance of wells treated with triton). [file 12936_2019_3071_MOESM1_ESM.tif]

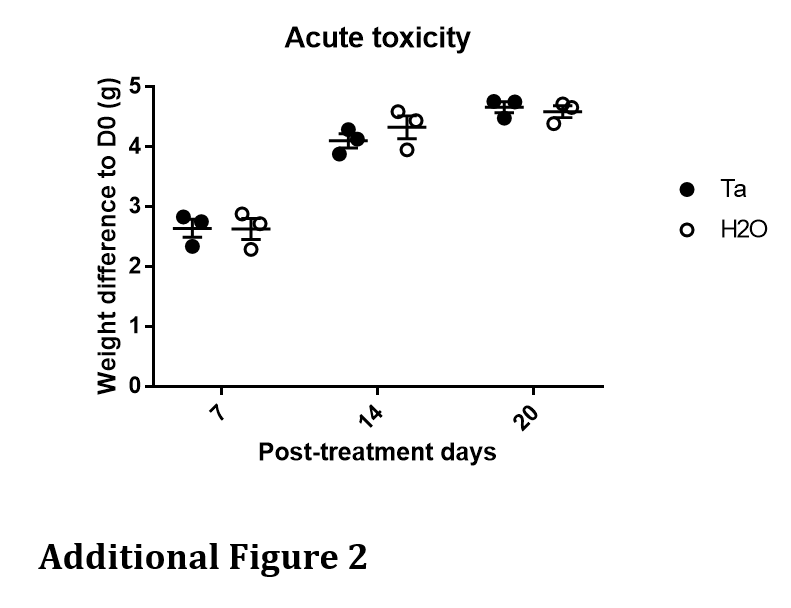

Supplement: Supplementary file 2 — Additional file 2: Fig. S2. Acute oral toxicity test in vivo: effect of Terminalia albida treatment on body weight. Mice were treated by oral route with a single dose of Terminalia albida (2000 mg/kg) or water (20 mL/kg). To evaluate the effect of Terminalia albida treatment on body weight, the weight was taken at D7, D14 and D20 and compared to D0 for both groups (Terminalia albida and water). [file 12936_2019_3071_MOESM2_ESM.tif]
